# Quantitative connection between polyglutamine aggregation kinetics and neurodegenerative process in patients with Huntington’s disease

**DOI:** 10.1186/1750-1326-7-20

**Published:** 2012-05-14

**Authors:** Keizo Sugaya, Shiro Matsubara

**Affiliations:** 1 Department of Neurology, Tokyo Metropolitan Neurological Hospital, 2-6-1 Musashidai, Fuchu, Tokyo, 183-0042, Japan

**Keywords:** Huntington’s disease, Polyglutamine aggregation, SCA3, Stochastic kinetics model, Cumulative damage, One-hit model, Nucleated growth polymerization, Nucleation, Elongation

## Abstract

**Background:**

Despite enormous progress in elucidating the biophysics of aggregation, no cause-and-effect relationship between protein aggregation and neurodegenerative disease has been unequivocally established. Here, we derived several risk-based stochastic kinetic models that assess genotype/phenotype correlations in patients with Huntington’s disease (HD) caused by the expansion of a CAG repeat. Fascinating disease-specific aspects of HD include the polyglutamine (polyQ)-length dependence of both age at symptoms onset and the propensity of the expanded polyQ protein to aggregate. In vitro, aggregation of polyQ peptides follows a simple nucleated growth polymerization pathway. Our models that reflect polyQ aggregation kinetics in a nucleated growth polymerization divided aggregate process into the length-dependent nucleation and the nucleation-dependent elongation. In contrast to the repeat-length dependent variability of age at onset, recent studies have shown that the extent of expansion has only a subtle effect on the rate of disease progression, suggesting possible differences in the mechanisms underlying the neurodegenerative process.

**Results:**

Using polyQ-length as an index, these procedures enabled us for the first time to establish a quantitative connection between aggregation kinetics and disease process, including onset and the rate of progression. Although the complexity of disease process in HD, the time course of striatal neurodegeneration can be precisely predicted by the mathematical model in which neurodegeneration occurs by different mechanisms for the initiation and progression of disease processes. Nucleation is sufficient to initiate neuronal loss as a series of random events in time. The stochastic appearance of nucleation in a cell population acts as the constant risk of neuronal cell damage over time, while elongation reduces the risk by nucleation in proportion to the increased extent of the aggregates during disease progression.

**Conclusions:**

Our findings suggest that nucleation is a critical step in gaining toxic effects to the cell, and provide a new insight into the relationship between polyQ aggregation and neurodegenerative process in HD.

## Background

The various disease-specific proteins involved in the distinct polyglutamine (polyQ) diseases, such as Huntington’s disease (HD) and spinocerebellar ataxia (SCA)-3 share no sequence homology, except in the polyQ tract. However, a strong and consistent inverse correlation has been found between the length of the expansion and the age of disease onset [1,2].

In vitro, aggregation of polyQ peptides follows a simple nucleated growth polymerization pathway [3]. Nucleated growth polymerization is a two-stage process consisting of the energetically unfavourable formation of a nucleus (i.e., nucleation), followed by efficient elongation of that nucleus via sequential addition of monomers [4]. Accordingly, the kinetics of the process feature a long lag time (nucleation lag time) followed by rapid aggregate growth. The detailed mechanism of nucleus generation based on polyQ sequences remains to be understood, however, the kinetic parameters of nucleation are expected to be exponential functions of repeat length [5].

Recent studies, however, have found multiple pathways underlying disease-related polyQ protein aggregation [6-8]. SCA3 is caused by expansion of the polyQ tract in ataxin-3 [9]. In vitro kinetic studies of ataxin-3 fibrillogenesis have revealed an alternative aggregation pathway in which full-length ataxin-3 has an intrinsic ability to form amyloid-like fibrils independent of the polyQ tract [6,10]. Remarkably, pre-fibrillar-like aggregates implicated in the toxicity of several neurodegenerative diseases are formed during the process of ataxin-3 aggregation [6,11]. These observations suggest the presence of a mechanism whereby expansion of the polyQ tract accelerates but is not structurally involved in misfolding of ataxin-3 into toxic intermediate structures.

One explanation of cell death in neurodegenerative disorders is that neurons gradually accumulate insults which ultimately overwhelm cellular homeostasis [12,13]. One mechanism frequently proposed to cause cumulative damage is oxidative stress [12,14], in which an imbalance between the production of reactive oxygen species and cellular antioxidant mechanisms results in chemical modifications of macromolecules, thereby disrupting cellular structures and functions. This scenario is supported by recent work in which the biophysics of aggregation were associated with the ‘toxic intermediate’ hypothesis, in which an imbalance occurs between the accumulation of toxic misfolded proteins, such as soluble oligomers/pre-fibrillar aggregates, and cellular protein quality control mechanisms [11,15].

Soluble oligomeric aggregate of mutant huntingtin (htt) protein in HD was also found in tissues from individuals with HD [16]. Some researchers have sought to advance cumulative-damage hypotheses seeking to explain HD pathogenesis [15,16]. In such scenarios, pathologic changes proceed at differential rates, depending on the length of expansion. This would result in disease onset when the changes were sufficiently severe to produce a disease symptom. The extent of expansion would then also govern the rate of clinical progression. However, it remains unknown whether the factors governing symptom onset also control clinical progression of HD. A large prospective study, assessed using clinical measures, suggested that polyQ-length had only a subtle effect on the rate of disease progression [17]. What mechanism of the disease progression might explain this discrepancy? Apparently, the cumulative-damage hypothesis cannot explain the exponential relationship between the extent of expansion and age at onset, nor the effect of polyQ-length on the progression of HD.

We first present two risk-based stochastic kinetic models to assess the genotype/phenotype correlations in patients with polyQ diseases. These models reflect the different disease pathomechanisms arising via two alternative aggregation pathways: the first is the cumulative-damage model, while the second is the one-hit model, wherein the one-hit event acts as a constant risk for neuronal cell damage and the kinetics of neuronal loss over time exhibit a first-order exponential function [18].

The stochastic appearance of nucleation of mutant htt proteins in a homogenous cell population over time could be expressed by a first-order exponential function [19]. If polyQ aggregate is actually related to disease pathogenesis, the probability of nucleation is considered to be a constant risk of neuronal damage regardless of toxicity because of the rate-limiting process of nucleation. In HD, the repeat-length dependent variability of age at onset could be reasonably explained by the length-dependent nucleation of polyQ aggregation kinetics [20]. However, we and others found that one additional polyQ-length independent factor also significantly contributes to the age at disease onset [20-22]. It is assumed that age-of-onset (*t*_A_) largely reflects a nucleation lag time (*t*_N_) and the additional time (_add_*t*) due to the polyQ-length independent factor. One-hit model of neurodegeneration can be improved with stretched-exponential decay models, which most easily fit data in which the rate of death decreases over time, are consistent with multiple populations of cells coexisting, each with a different constant rate of death [23]. However, even when we use a stretched-exponential decay model it alone is not enough to explain the genotype/phenotype correlations. By the regression analyses of polyQ-length versus age-of-onset in patients with HD, we further found that the sum of square relationship (*t*_A_^2^ = *t*_N_^2^ + _add_*t*^2^) showed the best fit among the models examined. This is well consistent with the relationship of tightly coupled processes of nucleation and elongation during disease progression. Under the assumption that *t*_N_ reflects the stochastic appearance of nucleation over time in a cell population, then it is reasonable to consider that _add_*t* reflects the distributed elongation times in the affected neurons (increased extent of aggregates as the nucleation events proceed). According to the hypothetical effect of aggregates on neuronal cell (protective or toxic), we finally derived three different models to examine whether polyQ aggregation kinetics by a nucleated growth polymerization mechanism can predict the effects of polyQ-length on the rate of disease progression as well.

## Methods

### Two risk-based stochastic kinetic models for neuronal cell loss

In inherited neurodegenerative disorders, delayed clinical onset (in which symptoms may not appear for years or decades) is often assumed to reflect the occurrence of age-dependent cumulative damage [24]. One prediction of the cumulative-damage hypothesis is that the probability of cell death will increase over time. However, Clarke et al. reported that the kinetics of neuronal death in many forms of neurodegeneration, including HD, appeared to be exponential, and in fact could be better explained by a mathematical model in which the risk of cell death remains constant (one-hit model). These models can be expressed by the risk-based stochastic kinetics as:

(1) $$\;dN\left(t\right)/dt=-r\left(t\right)\text{x}N\left(t\right)$$

where *N* is the number of remaining neurons, and *r*(*t*) represents the risk of cell death at age *t*[18]. Solving the differential equation of Eq. 1 generates an equation of exponential function as:

(2) $$N\left(t\right)/N\text{o}=\text{exp}[\int -r\left(t\right)dt]$$

where *N*_0_ is the number of neurons before neuronal cell death begins. The functions for *r*(*t*) were substituted as follows:

cumulative-damage model

(3) $$r\left(t\right)=r_{0}\text{exp}\left[A\left(t-t_{0}\right)\right]\;\left(A>0\right)$$

one-hit model

(4) $$r\left(t\right)=r_{0}\left(t>t_{0}\right)$$

where *r*_0_ represents the initial probability of cell death and *t*_0_ represents the time before neuronal death begins, and *r*_0_*e*^*A*^ corresponds to an increase in risk [18].

### PolyQ-length dependence of neuronal cell loss by a cumulative damage model

**Figure 1:**
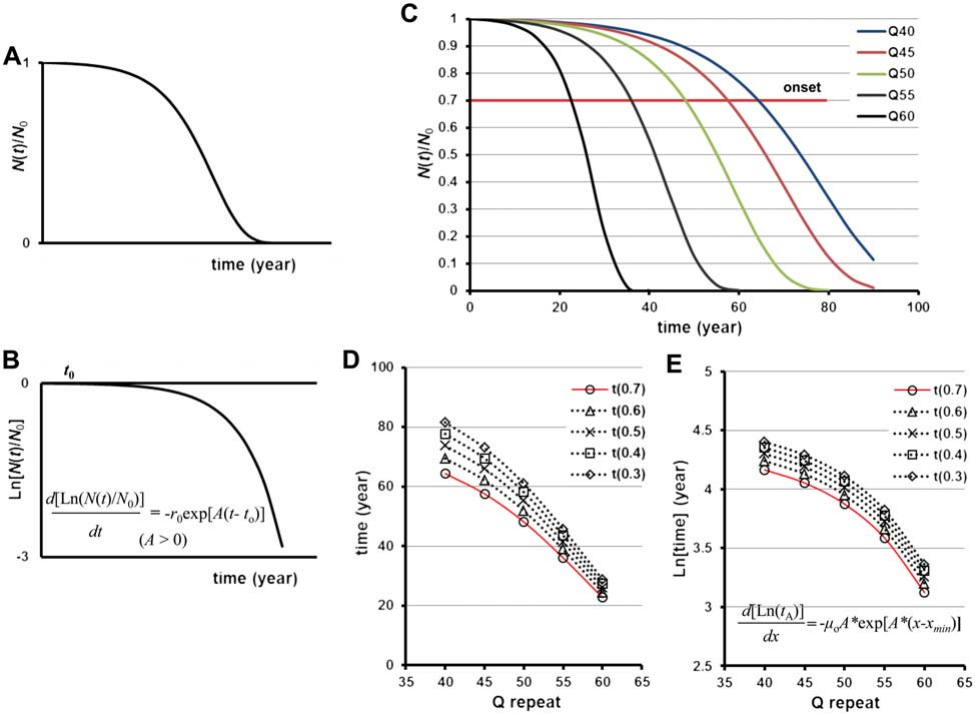
**Schematic representation of the kinetics of cell loss predicted by a cumulative-damage model.** (**A**) Ratio of intact neurons (*N*(*t*)/*N*_0_) as a function of time *t* shows a sigmoidal shape in the cumulative damage model (Eq. 2 and 3). (**B**) When displayed on a semi-log graph, neuronal survival curves over time, producing a shoulder shape. (**C-E**) PolyQ-length dependence of neuronal cell loss by the cumulative damage model. (**C**) Assuming that the extent of neuronal cell loss at the onset of disease is identical irrespective of polyQ-length (red line: 30% cell loss is defined as symptom onset), the relationship between polyQ-length and age-of-onset shows a sigmoidal function (Eq. 5b), and then the time course of neuronal cell loss for each polyQ-length should be described by sigmoidal kinetics, [as the disease proceeds, *t*(0.7) → *t*(0.3)]. (**D**) Relationship between polyQ-length and age at an identical ratio of cell loss for each polyQ-length shows a sigmoidal shape. Red line: age at onset for each polyQ-length [30% neuronal loss, *t*(0.7)]. (**E**) Semi-log plots of polyQ-length versus age at an identical ratio of cell loss for each polyQ-length show that the risks of cell death are represented by the correlation between polyQ-length and age-of-onset (red line).

In the cumulative-damage hypothesis, it was believed that there was only a small chance of a cell containing damage sufficient to initiate apoptosis early in the course of disease, with a correspondingly low rate of cell loss during this period. As the amount of intracellular damage increased with time, however, the chance of cell death also increased. The cumulative-damage model predicts that the neuronal survival curve will have a sigmoidal shape (Figure 1A), and that the risk of neuronal death will increase exponentially over time (Figure 1B) [18].

Under cumulative-damage conditions, polyQ-length dependence of the age-of-onset suggests that the risk of neuronal death increases exponentially with increased repeat length. Therefore, the relationship between polyQ-length and age-of-onset would be expected to be sigmoidal in nature, and may be described by Eq. 3 (with *t* substituted to *x*, and *N*(*t*)/*N*_0_ substituted to *t*_A_/*e*^*q-μ*o^) as follows:

(5) $$\text{Ln}(t_{\text{A}}/e^{q}{{}^{-\mu }}^{\text{o}})=-\int r\left(x\right)dx=-\int r_{\text{o}}\text{exp}[A^{*}\left(x-x_{\text{min}}\right)]dx$$

where *t*_A_ is age of disease onset, *x* is the repeat-length number, *x*_*min*_ is the minimum repeat length capable of causing neuronal death, *e*^*q-μ*o^ is the age of disease onset with *x* = *x*_*min*_, *μ*_o_ = *r*_o_/A^*^ and *r*_o_*e*^A*^ corresponds to an increase in risk with repeat number. Solving the integral equation of Eq. 5a generates an equation as:

(6) $$\text{Ln}\left(t_{\text{A}}\right)=q-\mu_{\text{o}}\text{exp}[A^{*}(x–x_{\text{min}})]$$

We initially made the reasonable assumption that the extent of neuronal cell damage at disease onset would be nearly identical irrespective of polyQ-length when the influence of normal aging was ignored. Then, Figure 1C-E shows an example of the cell-loss kinetics per polyQ-length based on the cumulative-damage model. If the relationship between polyQ-length and age-of-onset (red line) could be explained by a sigmoidal function, the relative time courses of neuronal cell damage for each polyQ-length would be expected to be described by a sigmoidal function (Figure 1C, D). As noted, in the sigmoidal function, the risk of neuronal cell death with time for each repeat length is precisely reflected by the correlation between polyQ-length and age-of-onset (red line in Figure 1E).

### PolyQ-length dependence of neuronal cell loss by a one-hit model

**Figure 2:**
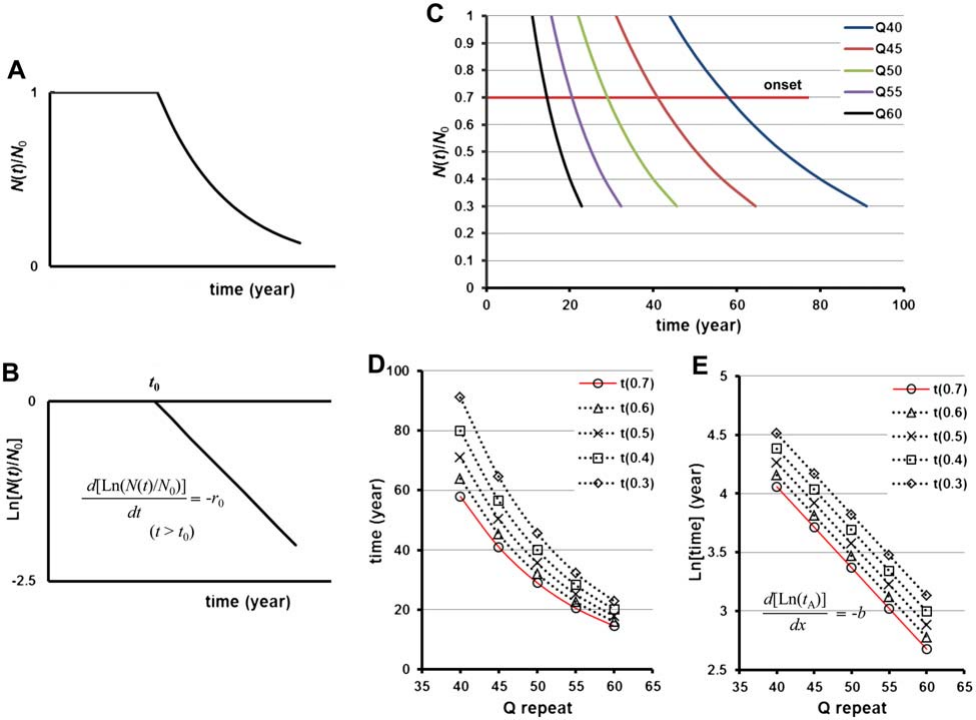
**Schematic representation of the cell loss kinetics predicted by a one-hit model.** (**A**) Ratio of intact neurons (*N*(*t*)/*N*_0_) as a function of time *t* shows an exponential decline in the one-hit model (Eq. 2 and 4). (**B**) When displayed on a semi-log graph, neuronal survival curves over time, producing a linear line. (C-E) PolyQ-length dependence of neuronal cell loss by the one-hit model. (**C**) Assuming that the extent of neuronal cell loss at the onset of disease is identical irrespective of polyQ-length (red line: 30% cell loss is defined as symptom onset), the relationship between polyQ-length and age of onset shows an exponential function (Eq. 6b), and then the time course of neuronal cell loss for each polyQ-length should be described by exponential kinetics, [as the disease proceeds, *t*(0.7) → *t*(0.3)]. (**D**) Relationship between polyQ-length and age at an identical ratio of cell loss for each repeat length shows an exponential shape. Red line: age at onset for each polyQ-length [30% neuronal loss, *t*(0.7)]. (**E**) Semi-log plots of polyQ-length versus age at an identical ratio of cell loss for each polyQ-length show that the slope (risks of cell death) are preserved during disease progression [*t*(0.7) → *t*(0.3)].

In a one-hit model, the kinetics of neuronal degeneration—i.e., the ratio *N*(*t*)/*N*_0_ of viable neurons as a function of time *t*—exhibited exponential cell loss decay (Figure 2A). Exponential kinetics, which can also be used to describe radioactive decay, indicate that the risk of cell death is constant (Figure 2B). In this process, the death of a neuron is initiated randomly in time by a single, rare catastrophic event [18].

Under this constant risk condition, the repeat-length dependence of the age-of-onset suggests that the probability of the rare event depends on the polyQ-length, and increases exponentially with repeat number. Thus, the relationship between repeat-length and age-of-onset (red line) would be expected to be exponential, described by using Eq. 2 and Eq. 4 (with *t* substituted to *x*, and *N*(*t*)/*N*_0_ substituted to *t*_A_/*a*) as:

(7) $$t_{\text{A}}/a=\text{exp}[-\int r\left(x\right)dx]=\text{exp}[-\int r_{\text{o}}dx]$$

Solving the integral equation of Eq. 6a generates an equation as:

(8) $$t_{\text{A}}=a\text{exp}[-b(x–x_{\min}{}_{*})]$$

where *x*_min*_ is the minimum repeat length capable of producing the rare catastrophic event, and *a* is the age of disease onset with *x* = *x*_min*_.

Figure 2C shows an example of the cell-loss kinetics per polyQ-length under the one-hit model. The relationship between polyQ-length and age of onset (red line) would be expected to be exponential. During disease progression, the probability of a one-hit event is distributed exponentially over time in keeping with the exponential correlation between the repeat-length and age-of-onset under the constant risk (red line, Figure 2D, E).

### PolyQ-length dependence of age at onset by a nucleation event

Exponential relationship between polyQ-length and nucleation lag time

An exponential relationship between the extent of expansion and the nucleation lag time was first proposed by Perutz et al. based on chemical thermodynamics [5]. The nucleation aggregation theory predicts that the probability of nucleation is an exponential function of the free energy of nuclear formation, which is proportional to exp[−*ΔG*_*crit*_/*kT*, where *ΔG*_*crit*_ is the critical free energy required to create a spherical nucleus, *k* is Boltzmann’s constant, and *T* is absolute temperature [5]. Since the addition of each glutamine stabilizes the helix structure by the formation of another three or four hydrogen bonds [25], the increase in free energy per additional repeat is expected to be constant [5]. The equation therefore predicts that the probability of nucleation rises exponentially with the number of repeats. The nucleation lag time is defined as the time required for the formation of a critical number of stable nuclei, leading to polymerization. If the minimum repeat length required to form stable nuclei is defined as *x*_*min*_^+^, thereby, when the conditions of monomeric nucleus, the nucleation lag time (*t*_N_) could be described using an exponential function of repeat-length, as:

(9) $$t_{\text{N}}=(N_{\text{cri}}/LC_{\text{mo}}R_{\text{o}})\text{xexp}[-b(x-x_{\min}{}^{+})](x≧x_{\min}{}^{+})$$

(10) $$\text{exp}[-b(x-x_{\min}{}^{+})]=\text{exp}[\Delta G_{+1}/kT+\Delta G_{+1}/kT+\Delta G_{+1}/kT+\Delta G_{+1}/kT\ldots ]$$

where *N*_cri_ is the critical number of nuclei required for polymerization in a given space, *L* is Avogadro’s number, *C*_mo_ is the bulk concentration of the expanded polyQ protein monomers, *R*_o_ is the initial rate of formation of stable nuclei, *x*_*min*_^+^ is the repeat length number in the first structure that is sufficiently stable to form a nucleus, *e*^*b*^ is the probability of nucleation at an individual repeat, and *ΔG*_+1_ is the change of free energy associated with one additional repeat.

Sum of square relationship among aggregation time, nucleation lag time and elongation time

An equation describing polyQ peptide aggregation kinetics in vitro by a nucleated growth polymerization mechanism was described by Chen et al. [3] as:

(11) $$\Delta =1/2\;k_{+}{}^{2}k_{\text{n}}C_{\mathrm{mo}}{}^{\left(\text{n}*+\;2\right)}t^{2}$$

where Δ is the concentration of monomer that has been incorporated into polymers, *k*_*+*_ is the forward elongation rate constant, *k*_n_ is the equilibrium constant describing the monomer-nucleus equilibrium, *C*_*mo*_ is the bulk concentration of monomers of the expanded polyQ peptides, n* is the critical nucleus (the number of monomeric units comprising the nucleus), and *t* is time. This equation represents the overall pathway of nucleated growth polymerization. Furthermore, the results from in vitro kinetic studies suggest that, in this equation, the only factor dependent on polyQ-length is the nucleation constant [3,26]. However, nucleation kinetics cannot be determined directly through physical measurement of nuclei because nucleation is a very rare event and the formed nuclei either quickly collapse to bulk phase monomer or proceed along the productive aggregation pathway [4]. Thus, instead of using polyQ aggregation kinetics to describe the overall pathway of nucleated growth polymerization, we derived a mathematical model that divides the time required for aggregation (*t*_agg_) into a polyQ-length-dependent nucleation lag time (*t*_N_), and a nucleation-dependent elongation time (*t*_E_). These were based on elongation kinetics obtained using aggregates of polyQ peptides as a seed; in these studies, the elongation rates were found to be nearly identical irrespective of polyQ-length [26-28] and aggregation rates were obtained to be *t*^2^-dependent [3,28]. Thus, the relationship could be approximated by Eq. 8 as:

(12) $$t_{\text{agg}}{}^{2}–t_{\text{E}}{}^{2}=t_{\text{N}}{}^{2}$$

Mathematical model describing the correlation between polyQ-length and age at onset

From Eq. 7 and Eq. 9, the following equation could be derived as:

(13) $${\left(t_{\text{agg}}{}^{2}–t_{\text{E}}{}^{2}\right)}^{1/2}=t_{\text{N}}=a^{*}\text{exp}[-b(x-x_{\min}{}^{+})]$$

where a^*^ is the nucleation lag time given the minimum repeat length (= *N*_cri_/*LC*_mo_*R*_o_). In agreement with a one-hit model by risk-based stochastic kinetics we demonstrated in a previous study that apart from SCA3, the relationship between polyQ-length and age-of-onset in all of the other polyQ diseases examined could be expressed by a first-order exponential function based on the repeat-length dependent nucleation of polyQ aggregation kinetics [20]. Assuming that polyQ aggregation is actually related to disease pathogenesis, we then hypothesized a mathematical model based on the assumption that the age-of-onset in HD largely reflects a nucleation lag time and the additional time. Using the large cohort of HD patients analyzed so far, Langbehn et al. found that the following exponential function provided excellent fits to both the mean and variance of the age of onset as:

(14) $$t_{\text{A}}–h=\text{exp}\left[i–jx\right]$$

where *hi* and *j* represent independent parameters, and *x* is the repeat number [21]. The principal cause of variability of age at onset is the length of polyQ repeats, however these findings suggest that the additional factor, which is independent of the polyQ-length, needs to be included to refine the model describing the correlation between polyQ-length and age at disease onset in HD. We further found that the following relationship between age-of-onset and the additional time (_add_*t*) due to the polyQ-length independent factor could be a better fit than Eq. 11:

(15) $${\left(t_{\text{A}}{}^{2}{–}_{\text{add}}t^{2}\right)}^{1/2}=\text{exp}\left[i–jx\right]$$

Eq. 12 is well consistent with Eq. 10. It is still unclear whether nucleation itself is toxic to neuronal cell. However, under the assumption that polyQ aggregation is related to HD pathogenesis because of the rate-limiting thermodynamically unfavourable state of nucleation, it is reasonable to consider that nucleation acts as a constant risk for neuronal cell damage based on Eq. 6b, Eq. 10, and Eq. 12. Therefore, Eq. 12 can be expressed as:

(16) $${\left(t_{\text{A}}{}^{2}{–}_{\text{add}}t^{2}\right)}^{1/2}=t_{\text{N}}=a^{*}\text{exp}[-b(x-x_{\min}{}^{+)}]$$

### Distribution function for nucleation lag time in a homogenous cell population

In Eq. 13, parameter *b*, which is a constant factor that depends only on polyQ-length, represents the nucleation rate with repeat number. Although many factors in this model are open to change, we can argue that if the environment remains relatively constant, only a few parameters will theoretically vary during disease progression. This suggests that in a homogenous cell population, the bulk concentration of the expanded polyQ protein monomers (*C*_mo_) and the additional time due to the polyQ-length independent factor are the principal contributors to the underlying pace of neurodegeneration, while the nucleation rate over time act as the constant risk of neuronal damage. This speculation is supported by the one-hit model (Figure 2) and by a previous study describing the aggregation behavior of mutant huntingtin htt proteins in a homogenous cell population [19]. Colby et al. found that in cultured striatal neurons, the probability of a cell remaining aggregate-free dropped exponentially with time at all examined expression levels of the mutant htt protein. Consistent with this exponential decay in the number of unaffected cells, a simple analytical theory exists for the lag time distribution for a stochastic nucleated polymerization reaction, in which the lag time of nucleation probability shown an exponential distribution [29]. After a period of nucleation initiation, the normalized probability distribution function for nucleation lag time can be simplified to [19]:

(17) $$P\left(t\right)=\varsigma \text{exp}[-\varsigma t]$$

where *ς* is the nucleation rate over time. Furthermore, the probability *P*_no agg_(*t*) that a given cell will be aggregate-free at time *t* will be given by [19]:

(18) $$P_{\text{no agg}}\left(t\right)=\int \varsigma \text{exp}{\left[-\varsigma t\right]}^{}dt=\text{exp}[-\varsigma t]$$

As the quantity of polyQ-expanded proteins in any one cell is likely to be too low to yield uniform aggregation behavior, nucleation is a rare event that occurs only in some cells, leading to the stochastic appearance of aggregate-containing cells [30]. The derivative equation describing the uniform aggregation behavior of polyQ-expanded peptides in vitro (Eq. 8) can be transformed as:

(19) $$\Delta =1/2\;k_{+}{}^{2}k_{n}C_{\mathrm{mo}}{}^{\left(\text{n}*+\;2\right)}t^{2}=\int k_{+}{}^{2}k_{n}C_{\mathrm{mo}}{}^{\left(\text{n}*+\;2\right)}tdt$$

The sum of the squared association between the times required for aggregation, nucleation lag, and elongation (*t*_agg_^2^ = *t*_N_^2^ + *t*_E_^2^) can be transformed as:

(20) $$\int t_{\text{agg}}=\int t_{\text{N}}+\int t_{\text{E}}$$

Thus, if the nucleation rate over time acts as a constant risk for neuronal cell damage in a homogenous cell population, the probability of aggregate-free neurons or the ratio of intact neurons (*N*(*t*)/*N*_0_) as a function of time *t* would be expected to decline exponentially, consistent with the stochastic appearance of nucleation. It is also assumed that nucleation and elongation act concomitantly during disease progression, in keeping with the relationship (*t*_agg_^2^ = *t*_N_^2^ + *t*_E_^2^).

### PolyQ-length dependence of neuronal cell loss by a stretched-exponential decay model

In a complex and heterogeneous population of cells involved in a specific brain lesion, multi-exponential decay functions can appear to provide a better fit for neuronal cell decay data under a one-hit model, compared to the assumption of mono-exponential decay. Indeed, this situation is better represented by the stretched-exponential function [23], which is a generalization of the exponential function with one additional parameter, the stretching exponent *β*, and is described by Eq. 2 and Eq. 4 as:

(21) $$N\left(t\right)/N_{\text{o}}=\text{exp}[-{\left(r_{\text{o}}\left(t-t_{\text{o}}\right)\right)}^{\beta }]$$

**Figure 3:**
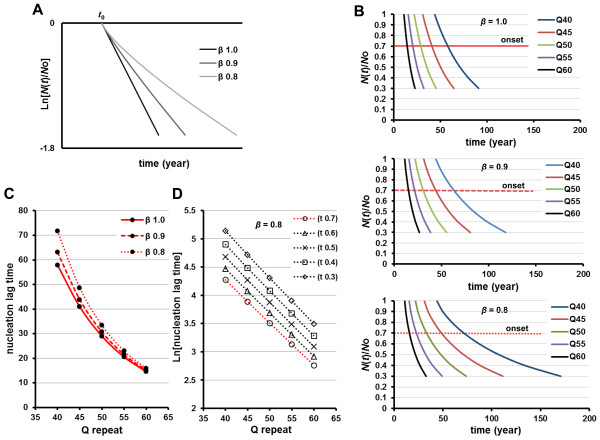
**Schematic representation of the cell loss kinetics predicted by stretched-exponential decay models.** (**A**) The stretched exponential is a generalization of the exponential function with one additional parameter: the stretching exponent, *β* (1 > *β* > 0). With *β* = 1, the simple exponential function (linearly on semi-log plots) is recovered (Eq. 16). The parameter *β* depends on the distribution width. (**B-D**) PolyQ-length dependence of neuronal cell loss by the stretched-exponential decay model. (**B**) Schematic representations of the cell-loss kinetics per polyQ-length in stretched-exponential functions with different *β* values (*β* = 1.0, 0.9, 0.8). Assuming that the extent of neuronal cell loss at the onset of disease (red line: 30% cell loss is defined as symptom onset) is identical irrespective of polyQ-length (**B**), the relationships between polyQ-length and nucleation lag time at disease onset, calculated by using Eq. 13 with different *β* values (*β* = 1.0, 0.9, 0.8), show a first-order exponential function regardless of the *β* values (**C**). (**D**) Semi-log plots of polyQ-length versus nucleation lag time at an identical ratio of cell loss for each polyQ-length (with *β* = 0.8) show that the slope of linear line (the risk of cell death) slightly changes during disease progression [*t*(0.7) → *t*(0.3)]. Red dotted line: nucleation lag time at disease onset for each polyQ-length [30% neuronal loss, *t*(0.7)].

The difference between the exponential function and the stretched-exponential function is schematically shown in Figure 3A. Figure 3B shows an example of the cell-loss kinetics per polyQ-length in a stretched-exponential decay model. Because the value of *β* is identical irrespective of polyQ-length, even in a stretched-exponential decay model, when neurodegeneration proceeds with a nucleation event as a constant risk of neuronal cell damage, the relationship between repeat-length and the nucleation lag time will be given by a first-order exponential function as shown in Eq. 13 regardless of the *β* value (Figure 3C, D). However, in contrast to the simple exponential function (homogeneous constant risk), the parameter of nucleation rate with repeat number (*b*) in Eq. 13 will change slightly during disease progression, thereby reflecting the coexistence of multiple population of neurons, each with a different constant rate of death (compare the slope of the linear lines in Figure 2E and Figure 3D). Thus, the additional time in Eq. 13 is not caused by reflection the coexistence of multiple populations of neurons. As noted, in the stretched-exponential function, polyQ-length affects the rate of disease progression more profoundly than the correlation between polyQ-length and age-of-onset, in inverse proportion to the stretching exponent, *β* (Figure 3B-D). These findings suggest that a stretched exponential decay model alone is not enough to explain the effects of polyQ-length on the rate of progression of HD, nor the correlation between the extent of expansion and age at disease onset.

### Striatal neurodegeneration in HD by a nucleated growth polymerization mechanism

Huntingtin is expressed in all cell types so far tested [31]. However, the most vulnerable cells to the toxic effect of the mutation are the neurons in the striatum. There is a marked selective pattern of neuronal degeneration within the striatum. Medium-sized, spiny projection neurons are disproportionately affected early and most severely in the disease, while large and medium-sized aspiny interneurons are relatively spared. Volume changes of the caudate nucleus accurately predicted the onset of symptoms and are significantly correlated with disease progression [32]. Moreover, the age of onset and CAG repeat length are significantly correlated with the extent of striatal atrophy [33,34]. Already, 10 years before the onset of the clinical symptoms, a significant neuronal loss is observed [35]. The loss of medium-sized spiny neurons in the caudate nucleus thus reflects the symptom onset and disease progression. These findings suggest that the correlation between polyQ-length and age of HD onset is mainly governed by the time required for a particular neuronal loss (per Q repeat) in the nucleus and support the legitimacy of modeling approach to predict the time course of neurodegeneration in the striatum.

From the feature of stochastic aggregation behaviour (Eq. 15) and the relationship between nucleation lag time and age-of-onset (Eq. 13), if polyQ aggregation is actually related to disease pathogenesis, the time course of neuronal loss in the striatum is expected to be a better fit with a stretched exponential function (Eq. 16). However the effects of the polyQ-length independent factor (_add_*t* in Eq. 13) on neural cell damage produce subtle differences. Thus, Eq. 16 can be transformed as:

(22) $$N\left(t\right)/N_{\text{o}}=\text{exp}[-{\left(\varsigma^{*}\left(t_{\text{N}}-t_{\text{No}}\right)\right)}^{\beta }]=F\left({}_{\text{add}}t\right)$$

(23) $$t^{2}=t_{\text{N}}{}^{2}+{\;}_{\text{add}}t^{2}$$

where ς^*^ represents nucleation rate-associated constant described by Eq. 15, *t*_N0_ represents the time before a series of nucleation events appears, and *F*(_add_*t*) represents the function of the additional time due to the polyQ-length independent factor.

The sum of square relationship in Eq. 17 (*t*^2^ = *t*_N_^2^ + _add_*t*^2^) is well consistent with the relationship of tightly coupled processes of nucleation and elongation during disease progression. In some neurons, polyQ aggregates can be detected prior to the onset of symptom of the disease. Under the assumption that *t*_N_ reflects the stochastic appearance of nucleation over time in a cell population, then it is reasonable to consider that _add_*t* in Eq. 17 reflects the distributed elongation time in the affected neuron (increased extent of aggregates as the nucleation events proceed). This allows us to examine whether polyQ aggregation kinetics by a nucleated growth polymerization mechanism can reasonably explain the correlations between genotypes and phenotypes, including the age of onset and the rate of disease progression. According to the hypothetical effect of aggregates on neuronal cell (protective or toxic), we further introduced the three different models (Model A-C) which may potentially describe the time course of neurodegeneration in the striatum. When _add_*t* in Eq. 17 was substituted to *t*_E_ (elongation time), from the value of *t*_E_^2^ at the onset of disease (*t*_E-onset_^2^), *t*_E_^2^ can be varied from 0 to *t*_E-onset_^2^ at the initiation of neuronal loss.

Model A (*t*_E_^2^ = 0 at the initiation of neuronal loss): The kinetics of neuronal cell loss in Model A is schematically shown in Additional file 1: Figure S1. Nucleation is sufficient to initiate neuronal cell loss in the striatum. The probability of nucleation over time acts as constant risk for neuronal damage (gray line), while elongation reduces the risk against nucleation in proportion to the increasing extent of aggregates during disease progression (pink line). The aggregates play a protective role against polyQ toxicity. Model A can be expressed as:

(24) $$N\left(t\right)/N_{\text{o}}=\text{exp}[-{\left(\varsigma^{*}\left(t_{\text{N}}-t_{\text{No}}\right)\right)}^{\beta }]=F\left(t_{\text{E}}\right)=1-{\left(k^{*}t_{\text{E}}{}^{2}\right)}^{\beta }$$

(25) $$t^{2}=t_{\text{N}}{}^{2}+t_{\text{E}}{}^{2}$$

where k^*^ is the elongation rate-associated constant.

Model B (*t*_E_^2^ = *t*_E-onset_^2^ at the initiation of neuronal loss): The kinetics of neuronal cell loss in Model B is schematically shown in Additional file 2: Figure S2. PolyQ aggregate itself is required to induce neuronal cell damage, and toxic to the cell in the striatum. Nucleation is a prerequisite for the formation of toxic aggregates. *F*(*t*_E_) = *t*_E-onset_^2^, constant value.

Model C (0 < *t*_E_^2^ < *t*_E-onset_^2^ at the initiation of neuronal loss): The kinetics of neuronal cell loss in Model C is schematically shown in Additional file 3: Figure S3. A specific conformation of polyQ aggregates is required to induce neuronal cell damage in the striatum. However, further growth in the extent of the aggregate reduces the risk of neuronal damage by that specific conformation. *F*(*t*_E_) (black line) is variable function that depends on the value of *t*_E_^2^ at the initiation of neuronal loss. The intermediate product of polyQ aggregates via a pathway of nucleated growth polymerization is toxic to the cell.

### Data collection

Clinical data from patients with mutations in relevant genes, including 308 patients with SCA3 and 312 patients with HD, were derived from earlier reports [36-40]. Patients with homozygous mutations were excluded.

To elucidate the rate of disease progression in SCA3, we used correlations between CAG repeat-length and quantified progression in brain atrophy as visualized by MR imaging. Abe et al., working with 30 patients with SCA3, reported significant correlations between the CAG repeat number and the extent of atrophy (divided by age at the time of examination) in the pontine tegmentum and midbrain [41]. To adjust for individual variations in the size of the skull, the anteroposterior diameters of the pontine tegmentum and midbrain were expressed as a ratio to the distance between the nasion and the inion. These ratios in each SCA3 patient were subtracted from the grand mean of the same ratio in all of the control cases as the degree of atrophy of the pontine tegmentum and midbrain. The cited authors found that there was no significant correlation between age and size of the anatomic structures of concern (no significant effect of normal aging on the atrophy).

To elucidate the effects of polyQ-length on the rate of disease progression in HD, we used the data of neuropathological change in a post-mortem study, where the extent of striatum cell loss divided by subject age was compared with CAG repeat-length [42], and also the observed relationship between repeat-length and the rate of functional decline, as assessed by Quantified Neurological Examination (QNE) scoring administered over time to patients in a large cohort study [17]. The QNE is an instrument for quantifying the number and severity of neurologic findings in HD [43]. Factor analysis of the QNE items revealed three subscales of highly correlated items: measurement of chorea, an eye movement subscale, and the Motor Impairment Scale. Motor Impairment scores are highly correlated with striatal atrophy in the course of disease. CAG repeat-length was significantly associated with the rate of progression of these clinical measures except chorea [17].

### Statistical analysis

We used the UNISTAT 5.6 statistical package for Windows (Unistat) for all analysis. To examine the association between age-of-onset and polyQ-length, we employed linear regression with logarithmic transformation of Eq. 5b and Eq. 13, thus invoking an intrinsically linear model, as follows:

(26) $$\text{Ln}[q–\text{Ln}\left(t_{\text{A}}\right)]=A^{*}x+\text{Ln}(\mu_{\text{o}})–A^{*}x_{\min}+\epsilon$$

(27) $$\text{Ln}{\left(t_{\text{A}}{}^{2}{–}_{\text{add}}t^{2}\right)}^{1/2}=-bx+\text{Ln}(a^{*})+{\mathrm{bx}}_{\min}{}^{+}+\epsilon$$

where *ϵ* represents residual error. Linear regression analysis was then applied to determine *q* or _add_*t*^2^ values by identifying the points at which the *R*^2^ were identical for a quadratic curve and a linear model with the best fitting a linear relationship. Models were evaluated using *R*^2^ numbers, the *F*-test, and analyses of residual error to test whether the assumptions of the regression are reasonably satisfied. The small number of individuals with the shortest and longest repeat sizes precluded rigorous statistical analysis of these patients. Had such an analysis been conducted, the data would have overwhelmed other information and thus impacted excessively on the model parameters.

### Estimating the effect of normal aging on neuronal loss in the caudate nucleus

We initially made the reasonable assumption that the extent of neuronal cell damage at the onset of disease was nearly identical irrespective of the polyQ-length when the influence of normal aging was ignored. If the time required for a particular neuronal loss in the caudate nucleus for each repeat length reflects a correlation between repeat length and the age of onset, and if such a correlation (polyQ-length versus nucleation lag time) were exponential in nature, eliminating the effect of age on neuronal loss in the caudate nucleus would be expected to yield a higher *R*^2^ value for the best fit of a linear relationship between the logarithm of *t*_N_ at the age of onset and polyQ size. We first hypothesized that the loss of neurons in the nucleus was 30% of baseline number at the onset of disease, irrespective of polyQ length. Then we examined the gradual effect of age on neuronal loss. The neuron decreased in a linear manner by 0.08 ~ 0.16% per year of normal aging, in the interval 20 ~ 30 years of age. From there, we determined individual *R*^2^ values for the correlations between the logarithms of *t*_N_ and polyQ size. Recent MR imaging studies of volumetric and diffusion tensor imaging (providing a quantitative assessment of the microscopic diffusion properties of water in living tissue) suggest that age-related degenerative change, which is thought to reflect neuronal loss in the caudate nucleus, could be well described by a linear function [44-46]. Therefore, we used a linear model.

## Results

### Correlation between polyQ-length and age of onset in patients with SCA3

**Figure 4:**
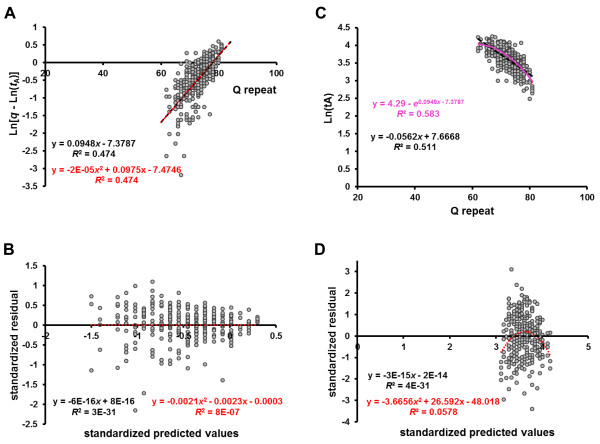
**Correlation between polyQ-length and age of onset in patients with SCA3.** A total of 308 patients with expanded CAG repeats in the ataxin-3 gene were analyzed. (**A**) Linear regression analysis using Eq. 19 with natural log-transformed [*q* – Ln(*t*_A_)] data against polyQ size provided the best fit to a linear model (0.0948*x* – 7.3787) when *q* = 4.29. The *q* value was determined by identifying the points at which the *R*^2^ were identical for a quadratic curve (red line) and a linear model (black line). (**B**) Residual analysis of standardized residuals versus standardized predicted values in the regression model of Figure 4A showed that the residual distribution was normal (i.e., mean = 0). Furthermore, the scattered points showed no clear pattern, suggesting that variance was constant. (**C**) Comparison of the regression model obtained from Figure 4A (pink line) to a linear regression of natural log-transformed *t*_A_ versus polyQ-length (black line). (**D**) Residual analysis of standardized residuals versus standardized predicted values in the regression model of natural log-transformed *t*_A_ versus polyQ-length showed an inverted U-shaped trend in the residual distribution (red line).

We previously reported a regression analysis of SCA3 disease onset against the CAG repeat lengths in the ataxin-3 genes of 308 patients, using a logarithmic transformation of age-of-onset (*t*_A_), and showed that it fit an inverted U-shaped curve [20]. This suggests that the relationship might be explained by a cumulative damage model in a risk-based stochastic kinetics (Figure 1B). Furthermore, recent in vitro studies of ataxin-3 aggregation kinetics support the cumulative-damage hypothesis in the pathogenesis of this disease [6]. We therefore performed a linear regression analysis using Eq. 19 (see Methods) with natural log-transformed *q* –Ln(*t*_A_)], and determined the *q* value (Figure 4A). The model was validated by a residual analysis (Figure 4B). We then compared the regression model (pink line) obtained from Figure 4A to a linear regression of natural log-transformed *t*_A_ versus polyQ-length (one-hit model, black line) (Figure 4C). Apparently, the regression analysis of the cumulative damage model using Eq. 19 (*R*^2^ = 0.583) showed a better fit than those of the one-hit model (*R*^2^ = 0.511) or a simple linear model (*t*_A_ versus polyQ-length, *R*^2^ = 0.515) (data not shown). As noted, the residual distribution of the regression by the one-hit model showed an inverted U-shaped trend (Figure 4D). The precise values of the parameters of interest, together with descriptive statistics, were determined by the regression analysis shown in Figure 4A, and the data are summarized in Table 1.

**Table 1:** **Parameters and descriptive statistics derived from the regression analyses of Figures **4**A and **6C

	**%patients**	**intercept ±SE**	**slope ±SE**	_**add**_***t***^**2**^	***q***	***R***^**2**^	***F*test**	***P***
SCA3	95.78	−7.3787 ± 0.424	0.0948 ± 0.00584	―	4.29	0.474	263.53	< 1.0 x 10^-41^
HD	98.42	6.6856 ± 0.144	−0.0668 ± 0.00318	217	―	0.588	441.47	< 1.0 x 10^-60^

SCA3: Ln[*q* –Ln(*t*_A_)] = A^*^x + Ln(*μ*_o_) – A^*^x_*min*_ + *ϵ* (Eq. 19).

HD: Ln(*t*_A_^2^ - _add_*t*^2^)^1/2^ = −*bx* + Ln(a^*^) + *bx*_*min*_^+^ + *ϵ* (Eq. 20).

% patients = percentage of all studied patients used to derive the model, SE = standard error, *R*^2^ = coefficient of determination. Clinical data from patients with mutations in relevant genes, including 308 patients with SCA3 and 312 patients with HD, were derived from earlier reports [36-40].

### Progression of illness in SCA3 patients may be expressed by the cumulative damage

**Figure 5:**
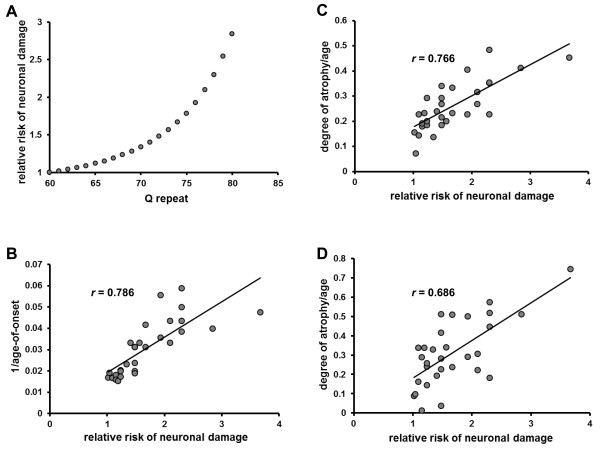
**Progression of illness in SCA3 patients may be expressed by a sigmoidal function.** (**A**) The relative risk of neuronal damage for each polyQ-length was derived from the regression model shown in Figure 4A. The risk of neuronal damage with a repeat number of 60 was defined as 1, and the relative risk of neuronal damage with a repeat number of 65 was calculated as age of onset for 60 repeats/age of onset for 65 repeats. (**B-D**) Data on the correlation between polyQ-length and quantified changes in brain atrophy as visualized by MR imaging [41] were re-evaluated using the relative risk of neuronal damage based on polyQ size. (**B**) The age of onset in patients analyzed by MR imaging was transformed as 1/age of onset, and correlated with the relative risk of neuronal damage based on polyQ size. Linear correlations between the relative risks of neuronal damage based on polyQ size and the age-adjusted degree of atrophy in the pontine tegmentum (**C**) and midbrain (**D**).

The relationship between polyQ-length and age-of-onset could be explained by a sigmoidal function. As the kinetics are sigmoidal, the relationship between progression of illness in SCA3 patients and polyQ-length is expected to be accurately reflected by the association between age-of-onset and the repeat-length (Figure 1E). By the regression model of Figure 4A, the relative risks of neuronal cell damage for each polyQ-length were calculated as shown in Figure 5A. Abe et al., working with 30 patients with SCA3, reported significant correlations between the CAG repeat number and the extent of atrophy (divided by age at the time of examination) in the pontine tegmentum and midbrain [41]. Pearson’s correlation coefficients were *r* = −0.88 for CAG size versus the age-of-onset, and *r* = 0.768 and *r* = 0.641 for CAG size versus the age-adjusted extent of atrophy in the pontine tegmentum and midbrain, respectively [41]. Here, these data were re-evaluated using the relative risk of neuronal cell damage based on polyQ size. The age of disease onset in each of these 30 patients was transformed as 1/age-of-onset, which corresponds to the rate of atrophy at the disease onset because of our assumption that the degree of neuronal damage at disease onset is nearly identical irrespective of polyQ-length when the influence of normal aging was ignored (Figure 1C). The correlation between these figures and the relative risk of neuronal damage based on polyQ size is shown in Figure 5B (*r* = 0.786, *p* < 0.0001). Significant correlations between the relative risk of neuronal damage based on polyQ size and the age-adjusted degree of atrophy in the pontine tegmentum (*r* = 0.766, p < 0.0001) and midbrain (*r* = 0.686, p < 0.0001) were observed (Figure 5C and D, respectively). The similarity between Figure 5B and C is striking (*r* = 0.786 versus 0.766), indicating that the relationship between the progression of atrophy in the pontine tegmentum and polyQ-length is precisely reflected by the association between age-of-onset and polyQ-length. As noted, simple linear correlation using Pearson’s correlation coefficient showed a slight difference between polyQ size versus age-of-onset and polyQ size versus age-adjusted degree of atrophy in the pontine tegmentum (*r* = 0.88 versus 0.768), but the sigmoidal relationship of the relative risk of neuronal damage to polyQ-length identified that the correlation was indeed very high (*r* = 0.786 versus 0.766). These findings suggest that disease progression may be kinetically sigmoidal in nature, and provide support for the cumulative-damage hypothesis in explaining the pathogenesis of SCA3.

### Correlation between polyQ-length and age of onset in patients with HD

**Figure 6:**
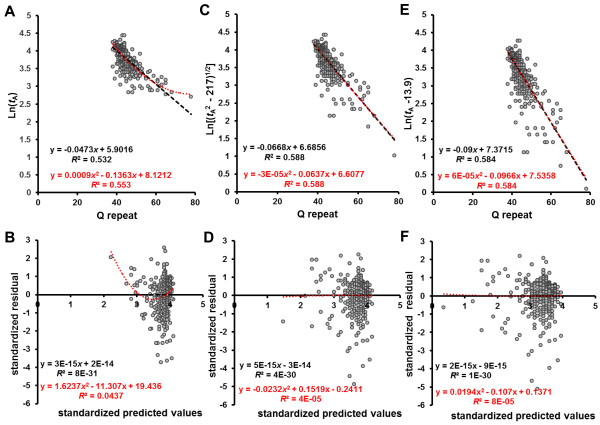
**Correlation between polyQ-length and age of onset in patents with HD.** A total of 312 patients with expanded CAG repeats in the HD gene were analyzed. (**A**) Linear regression analysis of natural log-transformed *t*_A_ versus polyQ size. (**B**) Residual analysis of standardized residuals versus standardized predicted values in the regression model of Figure 6A showed a U-shaped trend in the residual distribution. (**C**) Linear regression analysis using Eq. 20 with natural log-transformed (*t*_A_^2^ – _add_*t*^2^)^1/2^ data against polyQ size provided the best fit to a linear model when _add_*t*^2^ = 217. The _add_*t*^2^ value was determined by identifying the points at which the *R*^2^ were identical for a quadratic curve (red line) and a linear model (black line). (**D**) Residual analysis of standardized residuals versus standardized predicted values in the regression model of Figure 6C showed that the residual distribution was normal. (E) Linear regression analysis using Eq. 11 (see Methods) with natural log-transformed (*t*_A_ – *h*) versus polyQ-length to provide the best fit to a linear model when *h* = 13.9. (F) Residual analysis of standardized residuals versus standardized predicted values in the regression model of Figure 6D showed that the residual distribution was normal.

A linear regression analysis of natural log-transformed *t*_A_ versus polyQ-length in a total of 312 patients with HD is shown in Figure 6A (*R*^2^ value = 0.532). In contrast to SCA3, the residual distribution of the regression model showed a U-shaped trend (Figure 6B). We then performed a linear regression analysis using Eq. 20 (see Methods) with natural log-transformed (*t*_A_^2^ – _add_*t*^2^)^1/2^, and determined the _add_*t*^2^ value (Figure 6C). The model was validated by residual analysis (Figure 6D). We also examined a regression analysis using Eq. 11 by Langbehn et al. [Ln(*t*_A_ – *h*) versus polyQ size] to provide the best fit to a linear model when *h* = 13.9, yielding *R*^2^ value = 0.584 (Figure 6E). The model was also validated by residual analysis (Figure 6F). The regression analysis made using Eq. 20 provided the best fit to a linear model when _add_*t*^2^ = 217, yielding a highest *R*^2^ value (= 0.588) among the regression analyses. The principal cause of variability of age at onset is the length of the polyQ repeat. However, these findings confirmed the existence of the additional time due to the polyQ-length independent factor (_add_*t*^2^), which significantly contributes to the age of onset in patients with HD [20-22]. The sum of square relationship (*t*_A_^2^ = *t*_N_^2^ + _add_*t*^2^) showed the best fit among the models examined. The precise values of the parameters of interest, together with descriptive statistics, were determined by the regression analysis shown in Figure 6C, and the data are summarized in Table 1.

### Progression of illness in HD patients can be expressed by the nucleated growth polymerization mechanism

We derived three different models (Model A-C) which may potentially describe the time course of neurodegeneration in the striatum (see Methods), and examined whether polyQ aggregation kinetics by a nucleated growth polymerization mechanism can explain the effects of polyQ-length on the rate of disease progression as well.

By longitudinal analysis of caudate volume visualized by MR imaging in 40 HD gene-positive individuals, Hobbs et al. found that a difference in caudate volume between HD patients and controls was evident 14 years before motor disease onset, at which time the caudate volume was about 30% lower than the baseline value [45]. Around 20-30% of neurons within the caudate may already be lost prior to the onset of any motor related symptoms of the disease [47]. Therefore, we first hypothesized that neuronal loss in the caudate nucleus commences 14 years prior to disease onset, and that 30% of neurons within the caudate is decreased from the baseline at symptom onset. Because there is a markedly selective pattern of loss for the striatal neurons, we also first considered that the *β* value in Eq. 17 and Eq. 18 is close to 1 (the susceptibility of the affected neurons to polyQ toxicity is nearly homogenous, mono-exponential decay [18,48]). Thereby, if the influence of normal aging was ignored, the slope of linear regression line (Eq. 20) derived from the regression analysis of the correlation between polyQ-length and age-of-onset is expected to be identical during disease progression (Figure 2E). The time course of neuronal loss in the caudate nucleus can be estimated by the regression model. We also estimated the effects of normal aging on neuronal loss in the caudate nucleus (see Methods).

When we used Model A to estimate the effect of age on neuronal loss in the caudate nucleus (i.e., after the regression model (Figure 6C) was adjusted to reflect a decrease with neuronal cell number by 0.15% per year of normal aging from the age of 25 years), the regression analysis using Eq. 20 with natural log-transformed *t*_N_ values against polyQ size provided the best fit to a linear model (red arrow in Additional file 1: Figure S 1) when *t*_E_^2^ was 217, yielding the highest *R*^2^ value of 0.592 (data not shown). We calculated the value of *t*_No_ in Eq. 18 in accordance with model A. The value of ς^*^ in Eq. 18 was obtained for each polyQ-length using the values for *t*_N0_ (blue arrow) and *t*_N_ at the onset of disease (red arrow) (Additional file 1: Figure S 1). The time course of neuronal loss for each repeat-length was calculated and then recalibrated to reflect the influence of normal aging.

**Figure 7:**
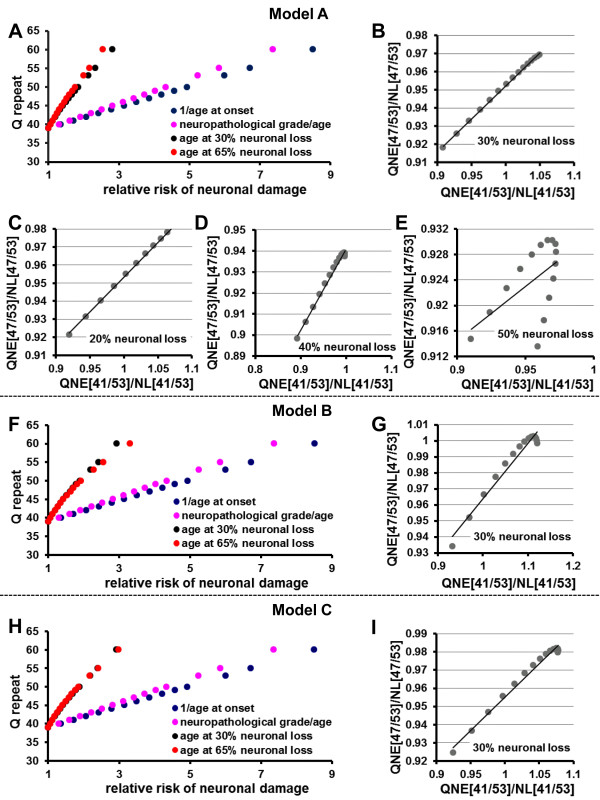
**Validation of the time course of neuronal loss in the caudate nucleus using Model A-C.** (**A, F, H**) When the risk of neuronal damage with a repeat number of 39 was defined as 1, the relative risks of neuronal damage were obtained from the correlation of polyQ size versus post-mortem striatal cell loss divided by subject age (pink circles) or by the correlation of polyQ size versus 1**/**reciprocal age at onset (blue circles) [42]. Using the time courses of neuronal loss estimated from each model, the relative risks of neuronal damage per polyQ-length were calculated by the age at 65% neuronal loss (red circles) and 30% neuronal loss (black circles) in a similar manner. (**B-E, G, I**) The predicted LOWESS plots of QNE scores versus disease duration for 41, 47 and 53 repeats were used [17]. The data on the estimated time courses of neuronal loss in each model were transformed to show the relationship between percentage of neuronal loss and disease duration from age at onset for patients with 41, 47 or 53 repeats. Here, the QNE scores and the percentage neuronal losses in the caudate nucleus against disease duration for 41 repeats or 47 repeats were divided by those for 53 repeats. Because QNE score is lineally increased by longer polyQ repeats during disease progression [17], we investigated the linear correlation between [(QNE scores at 41 repeats/QNE scores at 53 repeats) ÷ (neuronal losses at 41 repeats/neuronal losses at 53 repeats)] and [(QNE scores at 47 repeats/QNE scores at 53 repeats) ÷ (neuronal losses at 47 repeats/neuronal losses at 53 repeats)] (QNE[41/53]/NL[41/53] versus QNE[47/53]/NL[47/53]). We calculated the time courses of neuronal loss when neurons number account 30% of baseline at disease onset (B, G, I). In Model A, we also calculated when neurons number account 20 (C), 40 (D) or 50% (E) of baseline at disease onset.

When the risk of neuronal damage with a repeat number of 39 was defined as 1, the relative risk of neuronal damage per repeat-length is shown in Figure 7A, F and H. The degree of the effects of polyQ-length on the rate of neuropathological change (pink circle) is shown to be a lower than that on the age at disease onset (blue circle) [42]. Using the time courses of neuronal loss estimated from each model, we calculated the relative risks of neuronal damage for each repeat length, by the age at which there was a 30% neuronal loss (equivalent to age at disease onset, black circles), and a 65% neuronal loss (red circles) (Figure 7A, F and H). Because of the different models (linear versus exponential), the absolute values of the risk are different, however the time course of neuronal loss in Model A precisely reflected the relationship between the effect of polyQ-length on the age at disease onset and the effect of polyQ-length on the rate of neuropathological change (Figure 7A). We used the predicted LOWESS plots of the QNE scores versus disease duration for repeat numbers 41, 47 and 53 [17]. For comparison, we divided the QNE scores obtained for 41 repeats or 47 repeats by those obtained for 53 repeats (Figure 7B-E, G and I). Similarly, the estimated percentage of neuronal losses versus disease duration in each model for 41 repeats or 47 repeats was divided by that for 53 repeats (Figure 7B-E, G and I). The estimated rate of progression of neuronal losses in Model A for 41, 47 and 53 repeats precisely reflected the association between polyQ-length and clinical progression in HD patients (Figure 7B).

We also calculated the time course of neuronal loss in Model A when neurons losses account 20, 40 or 50% of baseline number at the onset of disease. Similarly, the estimated time courses of neuronal loss also precisely predicted the effects of polyQ-length on the rate of neuropathological change (data not shown). However, by comparing of the progression of QNE score against disease duration, we found that the estimated progression of neuronal loss in Model A precisely reflected the association between polyQ-length and clinical progression when we hypothesized neuronal loss account 20 or 30% of baseline at disease onset (Figure 7B-E).

Similarly, we calculated the time course of neuronal loss in Model B and Model C when neurons losses account 30% of baseline number at the onset of disease (Additional file 2: Figure S 2, Additional file 3: Figure S 3). In contrast to Model A, the time course of neuronal loss in Model B and Model C cannot reflect the effects of polyQ-length on the neuropathological change (Figure 7F, H), nor the association between polyQ-length and clinical progression assessed using QNE scoring (Figure 7G, I). In Model B and Model C, polyQ aggregate itself is thought to be required to initiate neuronal loss, and nucleation is a prerequisite for the formation of toxic aggregates. The time course of neuronal loss in Model C was calculated when *t*_E_^2^ at the initiation of neuronal loss = 100.

The rate of neuronal loss estimated using the mathematical model (Eq. 18) can precisely predict the correlation between repeat length and age of onset, as well as the association of repeat length with the rate of disease progression. These results suggest that nucleation is sufficient to initiate neuronal loss (Model A), and that around 20-30% of neurons within the caudate nucleus are lost at the onset of disease. However, our results cannot deny the possibility that a specific conformation of polyQ aggregates is required to induce neuronal cell damage (Model C). In Model C, *t*_E_^2^ at the initiation of neuronal loss is variable (0 < *t*_E_^2^ < 217). Then, Model C is nearly identical to Model A when the value of *t*_E_^2^ at the initiation of neuronal loss gets closer to 0.

The precise determination of the *β* value will be required for a large cohort-based longitudinal study in patients with the various CAG repeat length. Using Model A, we tested whether the *β* value is actually expected to be close to 1 or not. We examined the time courses of neuronal loss in the caudate nucleus using different values for the *β* parameter (*β* = 0.95, 0.9 and 0.8), and compared our results to the effects of polyQ-length on the rate of neuropathological change (Figure 7A). The relative risks of neuronal damage for each repeat-length were calculated in a manner similar to that shown in Figure 7A for the age at 30% neuronal loss and 65% neuronal loss (data not shown). Our results suggest the range of *β* value falls in a range of 1 > *β* > 0.95, and suggest that the susceptibility of the affected neurons to polyQ toxicity in the caudate nucleus is nearly homogeneous in the early and middle phases of HD. These results are well consistent with the reports by Miller et al. [49] using an automated microscope that tracked thousands of primary cultured striatal neurons individually over their entire lifetime. They found that until 6 days after the transfection of mutant HD gene, the kinetics of cell death showed a constant risk. This duration corresponds to the early and middle phases of the disease.

The function described by Langbehn et al. (Eq. 11) [21] also showed the excellent fit to the data obtained from the correlation between polyQ-length and age of onset in patents with HD (Figure 6E). However, using the regression model shown in Figure 6E, we found that the simple sum of relationship *t* = *t*_N_ + _add_*t* (*h*)] could not predict the effects of polyQ-length on the rate of disease progression in HD (data not shown).

## Discussion

Increased understanding of various polyQ diseases has revealed some common mechanistic features attributable to the existence of expanded polyQ tract *per se*[1,2,50]. However, recent studies have found multiple pathways underlying disease-related polyQ protein aggregation [6-8,51]. Tissues from individuals with HD contain mixtures of both elongation-competent and elongation-incompetent polyQ aggregates, suggesting the presence of multiple aggregates differing in both structure and toxicity [52]. Therefore, we must critically question whether alternative aggregation pathways contribute to possible differences in the mechanisms of polyQ disease pathogenesis.

Using polyQ-length as an index, we have here elucidated a relationship between aggregation pathways and disease onset or progression, and also an association between age at disease onset and rate of disease progression. Although disease mechanisms in polyQ disorders share unifying features, our modeling approach based on clinical data suggests that two different mechanisms may be involved in the pathogenesis of these conditions. Any quantitative connection between the relative risk of cell death for different repeat-lengths (as derived from the regression model for SCA3, Figure 5A), and a toxic effect of intermediate ataxin-3 aggregates, remains undetermined. However, our finding that correlations between genotypes and phenotypes, including both age at disease onset and rate of disease progression, could be reasonably explained using two distinct kinetic models corresponding to alternative pathways of aggregation, strongly support a central role for polyglutamine-mediated aggregation in disease pathogenesis.

Our results suggest that the time course of neurodegeneration in SCA3 may be represent by the cumulative damage model. There are many failure laws corresponding to cumulative damage for aging systems, and the most famous one in biology is the Gompertz law with exponential increase of the failure rates with age (Eq. 3), which is observed for many biological species including humans and is usually applicable within some age windows rather than the entire range of all possible ages [53,54]. The correlation between polyQ-length and age of onset in patents with SCA3 showed the best fit with the Gompertz function among the models examined (Figure 4). The other model describing the cumulative damage might be possible to be a better fit than the Gompertz function. However, it is required for a large cohort longitudinal study to determine whether such a model also show a better fit to the association between polyQ-length and the rate of disease progression. We also acknowledge that an essential condition for a cumulative damage model is that the rate of neurodegeneration is accelerated over years, but this was not shown in the present study. Compared to the other polyQ diseases, such an acceleration of disease progression in SCA3 was reported using clinical measure, but this study lack the relation to polyQ-length [55]. Very little longitudinal neuroimaging data are available for SCA3, and no study has examined the extent of neuronal cell damage at the onset of disease or its relationship (if any) to a particular brain structure. Further study are warranted, such a detailed longitudinal study that considers polyQ-length, including the effects of normal aging and clinical manifestations; this should confirm the sigmoidal kinetic nature of disease progression and clarify whether atrophy of the pontine tegmentum can be used as a biomarker of disease progression in SCA3.

In a one-hit model of neurodegeneration by Clarke et al., the death of a neuron is initiated randomly in time by a single, rare catastrophic event and the death of any given cell is independent of that of any other cell [18,56]. They further found that the one-hit model of neurodegeneration can be improved with stretched exponential decay models, which most easily fit data in which the rate of death decreases over time, are consistent with multiple populations of cells coexisting [23].

Our mathematical model (Eq. 18) describing the striatal neurodegeneration in HD consists with the one-hit model. In our model, polyQ-length dependent nucleation is considered to be a one-hit event. However, one important difference to the one-hit model exists. Using polyQ-length as an index, we demonstrated that even when we used a stretched-exponential decay model it could not fully explain the correlation between polyQ-length and age-of-onset in patients with HD, nor the association of polyQ-length with the rate of disease progression (Figure 3). By the regression analysis shown in Figure 6 and the modeling approach for the disease progression shown in Figure 7, we found that in addition to the nucleation event, the polyQ-length independent factor (_add_*t* in Eq.13 and 17) significantly contributes to the progression of neurodegenerative process. The data, moreover, suggest that its protective role against polyQ toxicity. Under the assumption that *t*_N_ in Eq. 17 reflects the stochastic appearance of nucleation over time in a cell population, then it is reasonable to consider that the polyQ-length independent factor reflects the distributed elongation times in the affected neurons because of the consistent figure of Eq. 10 and 13 and the relationship of tightly coupled processes of nucleation and elongation during disease progression. This allows us to examine whether polyQ aggregation kinetics by a nucleated growth polymerization mechanism could reasonably explain the genotype/phenotype correlations. However, the other scenario might be possible. For example, if dying neurons released a cyto-protective substance such as a neurotrophic factor into their environment and its protective effect can be expressed by the sum of square relationship as *t*^2^ = *t*_N_^2^ + _add_*t*^2^ (Eq.17), then the concentration of that factor will increase as more neurons affected, causing a concomitant decline in the risk of cell death. The functions in Eq. 18 (Model A) predict that the stochastic appearance of nucleation of mutant htt proteins acts as the constant risk of neuronal cell death over time, while elongation reduces the risk against nucleation in proportion to the increased extent of the aggregates during disease progression. Aggregate formation occurs via intrinsic properties of misfolded proteins, and is influenced by the environmental factors. Anyway, these scenarios suggest that although the initiation of the neurodegenerative process occurs randomly in time as a series of independent events for each neuron, the progression of the neurodegenerative process may be influenced by the other cells.

Using an automated microscope that tracked thousands of primary cultured striatal neurons individually over their entire lifetime, Miller et al. found that a specific monomeric conformer of the mutant htt protein strongly predicted neuronal cell death, and that increasing polyQ length preferentially increased the abundance of this conformer [49]. Nagai et al. analyzed the structural changes among purified polyQ protein in vitro and found that expanded polyQ proteins undergo a conformational transition to a cytotoxic *β*-sheet-dominant structure in the monomeric state [57]. In contrast to conventional models of nucleated growth polymerization, the in vitro aggregation kinetics of polyQ peptides show that the critical nucleus—the number of monomeric units comprising the nucleus—is equal to 1, suggesting that the rate-limiting nucleation process of polyQ aggregation is the folding of the mutated protein monomer [3]. Our modeling is compatible with the in vitro-derived aggregation kinetics of polyQ peptides, and demonstrates that nucleation is sufficient to initiate neuronal loss in the striatum. Taken together, these findings strongly suggest that nucleation is a critical step in gaining toxic effects to the cell. However, we cannot deny a principal pathogenic role for soluble oligomeric aggregates of mutant htt proteins, if they are made of a small number of monomeric units via a pathway of nucleated growth polymerization. In this case, we would expect the kinetics of cell death to closely fit Model A (corresponds to the situation where Model C is nearly identical to Model A when the value of *t*_E_^2^ at the initiation of neuronal loss gets closer to 0).

Arrasate et al. found that in cultured striatal neurons, among cells with comparable expression levels of the htt fragment, those that formed inclusion bodies were less likely to die than those that did not. The author suggested that inclusion body formation reduces the risk of neuronal death by decreasing the levels of toxic monomeric forms of mutant htt [58]. Our results well support these findings, although in our mathematical model, the risk of neuronal cell death decreases in proportion to increases in the extent of aggregates, rather than the formation of inclusion bodies itself. Thus, at least in the caudate nucleus, the pathological cellular effects of HD likely accrue via a pathway of nucleated growth polymerization, as represented by the mathematical model given in Eq. 18.

Toxic soluble oligomer/pre-fibrillar aggregates have been implicated in many other neurodegenerative conditions, including Alzheimer’s disease and Parkinson’s disease [11,59,60]. If, such aggregates play a major role in disease pathogenesis via a cumulative damage mechanism, we would expect that the time courses of neuronal cell damage would fit a sigmoidal function, as shown in the case of SCA3 (Eq. 3). However, for example, recent positron emission tomography studies of Parkinson’s disease have invariably shown exponential declines in nigrostriatal dopamine function over time [61-63], consistent with a one-hit mechanism of neurodegeneration. A heterogeneous population of soluble oligomeric species of α-synuclein (which is believed to be a major player in the pathogenesis of Parkinson’s disease) has been identified as forming via different aggregation pathways [64]. If toxic soluble oligomers occur via a pathway of nucleated growth polymerization, we would expect the kinetics of neuronal cell damage to closely fit an exponential function, as shown in the case of HD (Eq. 18). We propose, therefore, that at least these two distinct kinetic models, which are both based on the aggregation of misfolded proteins, could explain the gain of toxic functions in neurodegenerative disorders.

## Conclusions

Although disease mechanisms are superficially attributable to the existence of expanded polyQ *per se*, our findings suggest that two different mechanisms, reflecting alternative pathways of aggregation, may be involved in the pathogenesis of polyQ diseases. We successfully derived a quantitative connection between polyQ aggregation and neurodegenerative processes in HD, including the time of disease onset and the progression of illness in HD patients. Finally, we developed the mathematical model by which the time course of striatal neurodegeneration in HD can be precisely predicted. The results of our modeling approach suggest that neurodegeneration occurs by different mechanisms for the initiation and progression of disease processes. The repeat-length dependent one-hit event acts as a constant risk for neurodegeneration, while the repeat-length independent factor plays a significant protective role against the risk by the on-hit events in the progression of HD. Their relationship could be expressed by the sum of square relationship. These processes can be well explained by the nucleated growth polymerization mechanism of polyQ aggregation, providing a new insight into the framework for relating aggregation kinetics with molecular mechanism underlying neurodegenerative process.

## Competing interests

The authors declare that they have no competing interests.

## Authors’ contributions

Conceived and designed the models: KS. Analyzed the data: KS. Wrote the paper: KS SM. All authors read and approved the final manuscript.

## Supplementary Material

Additional file 1 **Figure S1. Schematic representation of the functions of Model A (Eq. 18) with *β * = 1.** Gray line reflects the probability distribution function for nucleation lag time (*t*_N_) against the number of unaffected cells (*N*(*t*)/*N*_0_) shows a first-order exponential function. Pink line represents the time course of neuronal loss in Model A. Black line: the function of elongation time versus *N*(*t*)/*N*_0_ [*F*(*t*_E_) in Eq. 18]. The co-relational model of polyQ-length versus *t*_N0_ (blue arrow) was derived from the regression model of the correlation between polyQ-length and age of onset in patients with HD (Figure 6C). After elimination of the estimated effect of normal aging on the caudate nucleus, the regression analysis of polyQ-length versus Ln(*t*_N_ at disease onset) provided the highest *R*^2^ value to the linear model (red arrow). Slope of the gray line (ς^*^ in Eq. 18) can be obtained for each polyQ-length using the values for *t*_N0_ and *t*_N_ at disease onset. Slope of the black line was obtained from the values for *t*_E_^2^ at the initiation of neuronal loss (= 0) and *t*_E_^2^ at disease onset (= 217). Click here for file

Additional file 2 **Figure S2. Schematic representation of the function of Model B (Eq. 17with**_**add **_***t *substituted to *t***_**E**_**, and *β* = 1).** Gray line reflects the probability distribution function for nucleation lag time (*t*_N_) against the number of unaffected cells (*N*(*t*)/*N*_0_) shows a first-order exponential function. Pink line represents the time course of neuronal loss in Model B. The elongation time (*t*_E_) is constant value (*t*_E_^2^ = 217). When we used Model B to estimate the effect of age on neuronal loss in the caudate nucleus (i.e., after the regression model (Figure 6C) was adjusted to reflect a decrease with neuronal cell number by 0.09% per year of normal aging from the age of 25 years), the regression analysis using Eq. 20 with natural log-transformed (*t*_A_^2^ – *t*_E_^2^)^1/2^ values against polyQ size provided the best fit to the linear model (red arrow) when *t*_E_^2^ was 217, yielding the highest *R*^2^ value of 0.591 (data not shown). We calculated *t*_N0_ in Eq. 17 (blue arrow) in accordance with model B. Then, the slope of gray line (ς^*^ in Eq. 17) can be obtained for each polyQ-length using the values for *t*_N0_ and *t*_N_ at disease onset. Click here for file

Additional file 3 **Figure S3. Schematic representation of the function of Model C (Eq. 17with**_**add **_***t * substituted to *t***_**E**_**, and *β* = 1).** Gray line reflects the probability distribution function for nucleation lag time (*t*_N_) against the number of unaffected cells (*N*(*t*)/*N*_0_) shows a first-order exponential function. Pink line represents the time course of neuronal loss in Model C. The elongation time (*t*_E_) at the initiation of neuronal loss is variable (0 < *t*_E_^2^ < 217). Here, the time course of neuronal loss in Model C was calculated when *t*_E_^2^ at the initiation of neuronal loss = 100. When we used Model C to estimate the effect of age on neuronal loss in the caudate nucleus (i.e., after the regression model (Figure 6C) was adjusted to reflect a decrease with neuronal cell number by 0.13% per year of normal aging from the age of 25 years), the regression analysis using Eq. 20 with natural log-transformed (*t*_A_^2^ – *t*_E_^2^)^1/2^ values against polyQ size provided the best fit to the linear model (red arrow) when *t*_E_^2^ was 217, yielding the highest *R*^2^ value of 0.593 (data not shown). We calculated *t*_N0_ in Eq. 17 (blue arrow) in accordance with model C. Then, the slope of gray line (ς^*^ in Eq. 17) can be obtained for each polyQ-length using the values for *t*_N0_ and *t*_N_ at the onset of disease. Click here for file
