# Supplementary figures and images for: Quantitative connection between polyglutamine aggregation kinetics and neurodegenerative process in patients with Huntington’s disease
Source: Mol Neurodegener. 2012 May 14;7:20. doi: 10.1186/1750-1326-7-20 (PMC3468392; doi:10.1186/1750-1326-7-20)

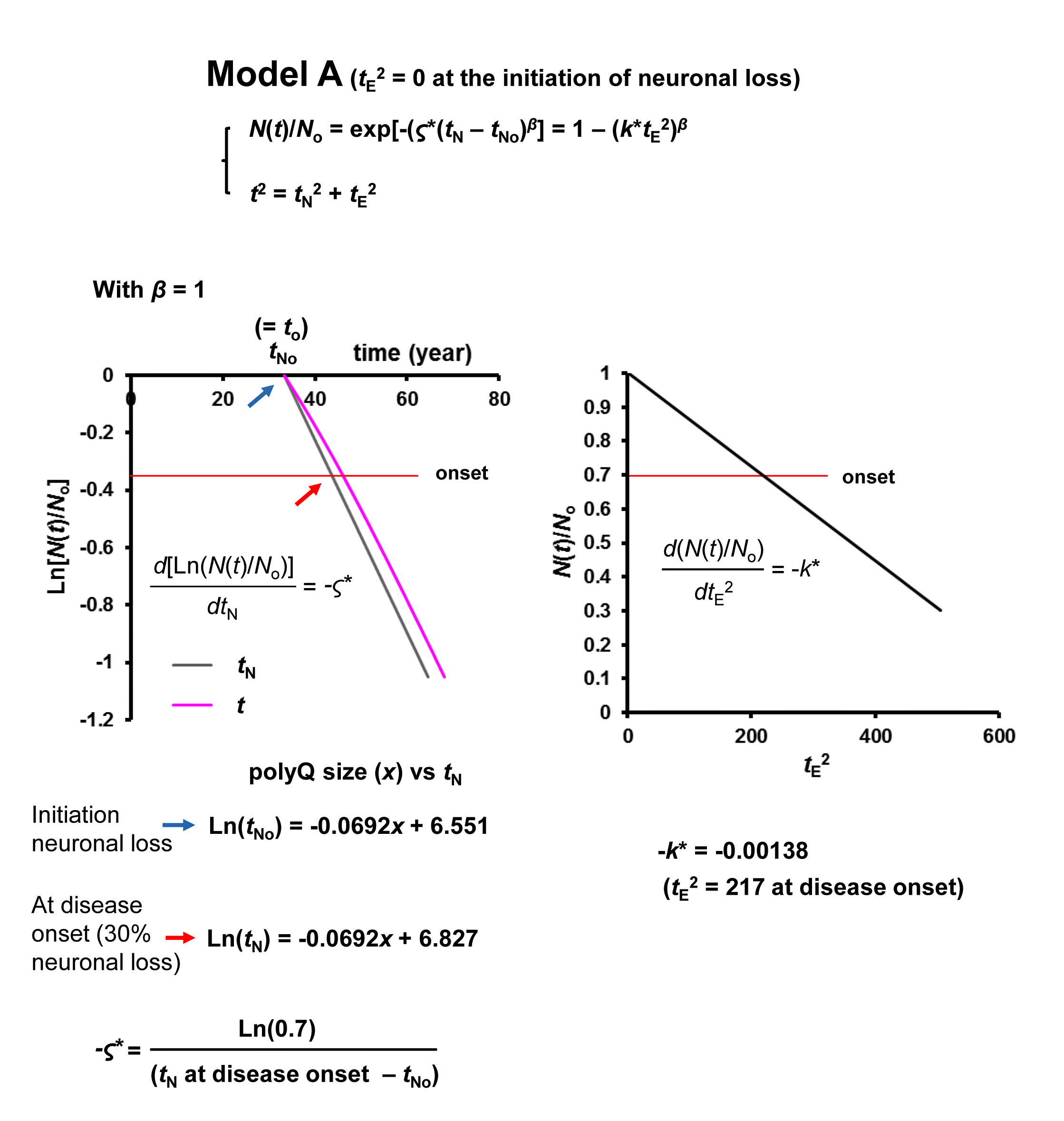

Supplement: Additional file 1 — Figure S1. Schematic representation of the functions of Model A (Eq. 18) with β = 1. Gray line reflects the probability distribution function for nucleation lag time (tN) against the number of unaffected cells (N(t)/N0) shows a first-order exponential function. Pink line represents the time course of neuronal loss in Model A. Black line: the function of elongation time versus N(t)/N0 [F(tE) in Eq. 18]. The co-relational model of polyQ-length versus tN0 (blue arrow) was derived from the regression model of the correlation between polyQ-length and age of onset in patients with HD (Figure 6C). After elimination of the estimated effect of normal aging on the caudate nucleus, the regression analysis of polyQ-length versus Ln(tN at disease onset) provided the highest R2 value to the linear model (red arrow). Slope of the gray line (ς* in Eq. 18) can be obtained for each polyQ-length using the values for tN0 and tN at disease onset. Slope of the black line was obtained from the values for tE2 at the initiation of neuronal loss (= 0) and tE2 at disease onset (= 217). [file 1750-1326-7-20-S1.tiff]

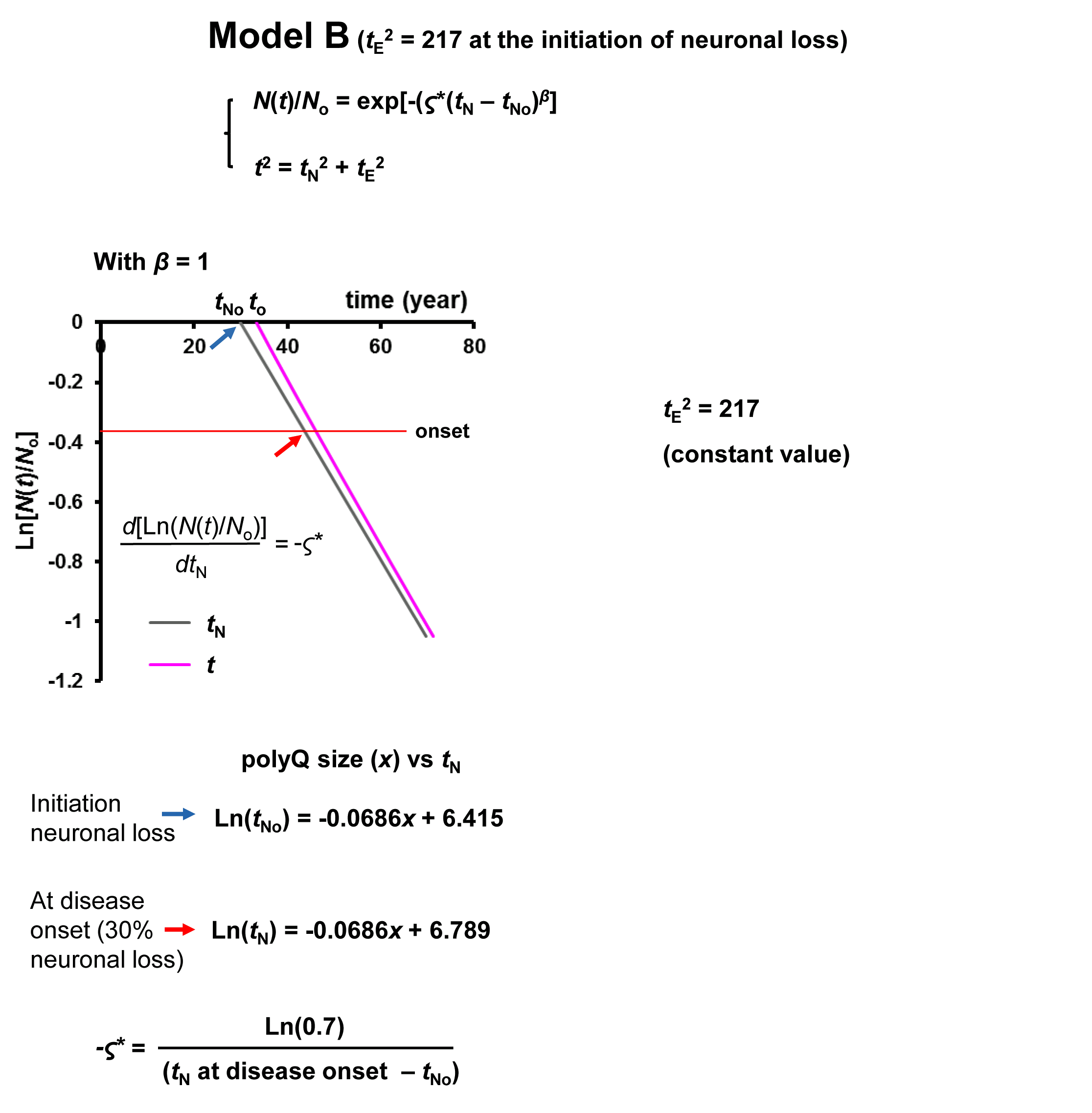

Supplement: Additional file 2 — Figure S2. Schematic representation of the function of Model B (Eq. 17withadd t substituted to tE, and β = 1). Gray line reflects the probability distribution function for nucleation lag time (tN) against the number of unaffected cells (N(t)/N0) shows a first-order exponential function. Pink line represents the time course of neuronal loss in Model B. The elongation time (tE) is constant value (tE2 = 217). When we used Model B to estimate the effect of age on neuronal loss in the caudate nucleus (i.e., after the regression model (Figure 6C) was adjusted to reflect a decrease with neuronal cell number by 0.09% per year of normal aging from the age of 25 years), the regression analysis using Eq. 20 with natural log-transformed (tA2 – tE2)1/2 values against polyQ size provided the best fit to the linear model (red arrow) when tE2 was 217, yielding the highest R2 value of 0.591 (data not shown). We calculated tN0 in Eq. 17 (blue arrow) in accordance with model B. Then, the slope of gray line (ς* in Eq. 17) can be obtained for each polyQ-length using the values for tN0 and tN at disease onset. [file 1750-1326-7-20-S2.tiff]

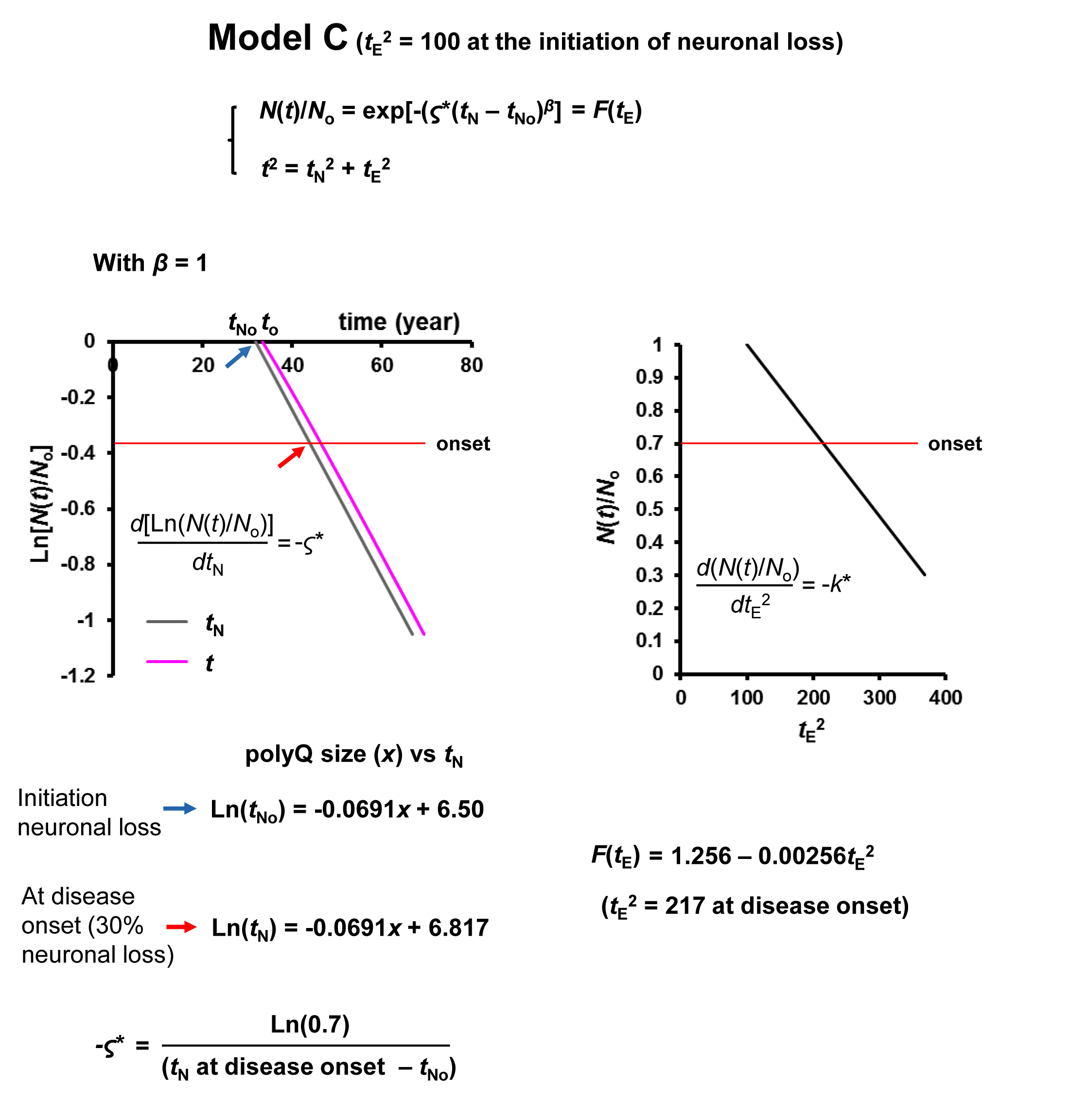

Supplement: Additional file 3 — Figure S3. Schematic representation of the function of Model C (Eq. 17withadd t substituted to tE, and β = 1). Gray line reflects the probability distribution function for nucleation lag time (tN) against the number of unaffected cells (N(t)/N0) shows a first-order exponential function. Pink line represents the time course of neuronal loss in Model C. The elongation time (tE) at the initiation of neuronal loss is variable (0 < tE2 < 217). Here, the time course of neuronal loss in Model C was calculated when tE2 at the initiation of neuronal loss = 100. When we used Model C to estimate the effect of age on neuronal loss in the caudate nucleus (i.e., after the regression model (Figure 6C) was adjusted to reflect a decrease with neuronal cell number by 0.13% per year of normal aging from the age of 25 years), the regression analysis using Eq. 20 with natural log-transformed (tA2 – tE2)1/2 values against polyQ size provided the best fit to the linear model (red arrow) when tE2 was 217, yielding the highest R2 value of 0.593 (data not shown). We calculated tN0 in Eq. 17 (blue arrow) in accordance with model C. Then, the slope of gray line (ς* in Eq. 17) can be obtained for each polyQ-length using the values for tN0 and tN at the onset of disease. [file 1750-1326-7-20-S3.tiff]
